# The Mitochondrial-Associated Endoplasmic Reticulum Membrane and Its Role in Diabetic Nephropathy

**DOI:** 10.1155/2021/8054817

**Published:** 2021-11-05

**Authors:** Lihua Ni, Cheng Yuan

**Affiliations:** ^1^Department of Nephrology, Zhongnan Hospital of Wuhan University, Wuhan 430071, China; ^2^Department of Gynecological Oncology, Zhongnan Hospital of Wuhan University, Wuhan 430071, China

## Abstract

The mitochondrial-associated endoplasmic reticulum membrane (MAM) is located between the outer mitochondrial membrane and the endoplasmic reticulum membrane. The MAM is involved in a wide range of cellular functions, including calcium signaling, the division and fusion of mitochondria, endoplasmic reticulum stress, and the synthesis and transport of lipids. Recent studies have discovered that the MAM is involved in the pathogenesis of diabetic nephropathy (DN). In this article, we summarize the structure, function and role of the MAM in DN. We hope this study will provide clues and a theoretical basis for mechanistic and targeted drug research on DN.

## 1. Overview of Diabetic Nephrology

The prevalence of diabetes mellitus (DM) is projected to increase rapidly in the upcoming decades and increase to 642 million by 2040 [1]. Diabetic nephropathy (DN) is one of the most frequent and serious chronic complications of DM. This microvascular complication occurs in approximately 30% of patients with type 1 DM (T1DM) and approximately 40% of patients with T2DM [2, 3]. According to epidemiological studies, DN is the leading cause of end-stage renal disease (ESRD) worldwide [3]. DN is a major but under-recognized contributor to the global public health burden. Several studies have shown that the 10-year mortality rates of patients with DN are equal to the average mortality rates of all cancers [4, 5]. Thus, there is a strong rationale to conduct research on DN.

The disease progression of DN includes glomerular hyperfiltration, progressive albuminuria, decreased GFR and finally ESRD. The pathological changes include glomerular hypertrophy, mesangial cell proliferation and hypertrophy, thickening of the glomerular and tubular basement membrane (GBM and TBM), glomerulosclerosis, tubulointerstitial inflammation and renal fibrosis.

The specific mechanism of DN has been widely studied. Generally, hemodynamic changes in the kidney, disorders of lipid metabolism, the inflammatory response, oxidative stress, endoplasmic reticulum stress and the formation of advanced glycosylation products are the leading causes and are involved in the pathogenesis of DN [6–9]. These processes contribute to the expansion of the mesangial matrix, Kimmelstiel-Wilson lesions, the thickening of GBM and TBM, podocyte injury and deletion, and interstitial fibrosis. It is worth mentioning that the onset of DN is considered to involve multiple factors rather than just a single factor. With the advancement of research, scholars have discovered that the mitochondria-associated endoplasmic reticulum membrane (MAM) also plays crucial roles in the progression of DN [10, 11].

## 2. Overview of the MAM

**Figure 1:**
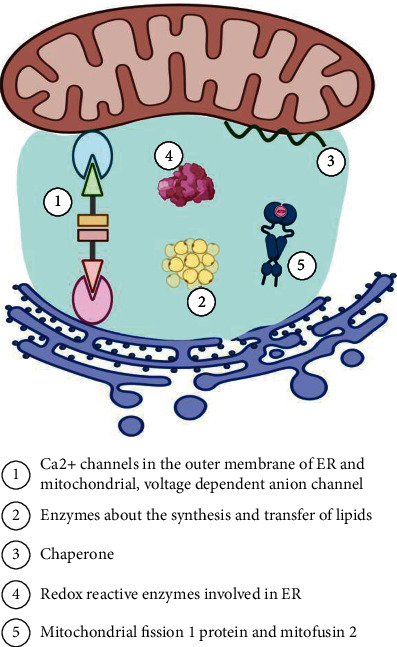
The structure and components of the mitochondrial-associated ER membrane. The physical structure between the ER and mitochondria is designated the MAM. Several enzymes, molecules and proteins are located in the MAM and participate in cellular activities, which can be summarized into five categories. Note: ER, endoplasmic reticulum; MAM, mitochondria-associated ER membrane.

As important organelles of eukaryotic cells, mitochondria and the endoplasmic reticulum (ER) are closely connected and function together in cellular activities in humans. Mitochondria are bioenergetic and biosynthetic organelles, which are also known as “power stations” [12]. They can provide a steady source of energy for human activities. Additionally, they also function as a biosynthetic platform for generating building blocks. In addition, the ER is defined as the “base station of protein and lipid synthesis”, as it is responsible for the synthesis, modification and processing of proteins and lipids [13, 14]. Interestingly, the ER is a dynamic intracellular organelle. The structure and function of the ER are not static during different cellular activities. Recent studies have shown that there are close structural and functional connections between mitochondria and the endoplasmic reticulum [15–17]. Through both fluorescence microscopy and electron microscopy, it has been observed that there are mutually coupled membrane components between these organelles [18], which has been designated as the MAM, shown in Figure 1. It is not surprising that this physical association promotes the interaction between the ER and mitochondria and participates in the regulation of cellular activities in humans.

## 3. MAM Discovery and Components

**Figure 2:**
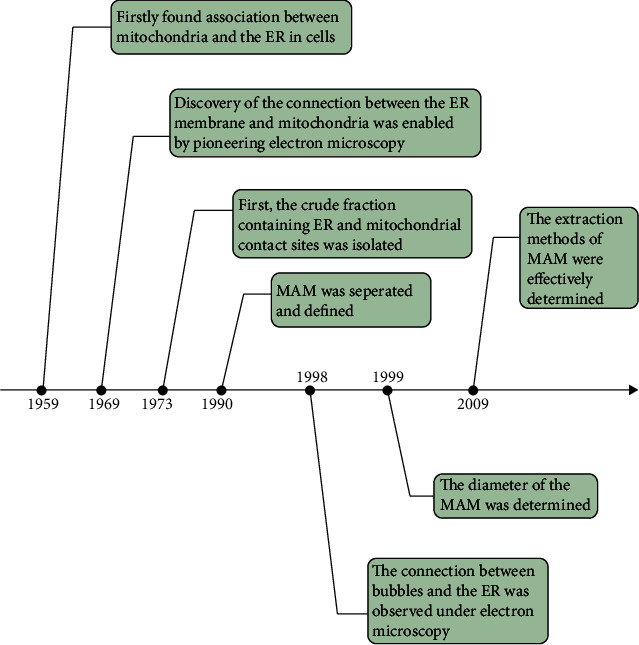
Historical timeline of the most important observations and findings related to the mitochondrial-associated ER membrane. Note: ER, endoplasmic reticulum; MAM, mitochondria-associated ER membrane.

The MAM is present in several cell types and consists of a small section of the outer mitochondrial membrane and the ER. Several studies have revealed that the MAM represents a morphological adaptation facilitating communication between the mitochondria and the ER (Figure 2). As early as 1959, Copeland and Dalton found an association between mitochondria and the ER in pseudobranch cells [19]. In 1969, Ruby et al. discovered continuities between the ER and mitochondria with a Philips 2000 electron microscope [20]. In 1973, Lewis et al. isolated the first crude fraction containing ER and mitochondria contact sites [21]. For the first time in 1990, Vance et al. separated the inner cell membrane structure in adsorbed hepatocytes and defined it as the MAM [22]. As research progressed, it was found that the MAM is involved in lipid metabolism [23–26]. With developments in electron microscopy technology, Mannella et al. observed the connection between bubbles and the endoplasmic reticulum via electron microscopy in 1998 [27]. In 1999, Achleitner et al. determined that the diameter of the connection between the endoplasmic reticulum and mitochondria ranges from 10 to 60 nm [28]. In 2009, Wiechowski et al. summarized an efficient extraction method for the MAM [29]. At present, there are an increasing number of studies on the MAM, and the composition of the MAM has been gradually described (Table 1). In the next section, we will focus mainly on the function and role of the MAM in DN.

## 4. MAM Function

**Figure 3:**
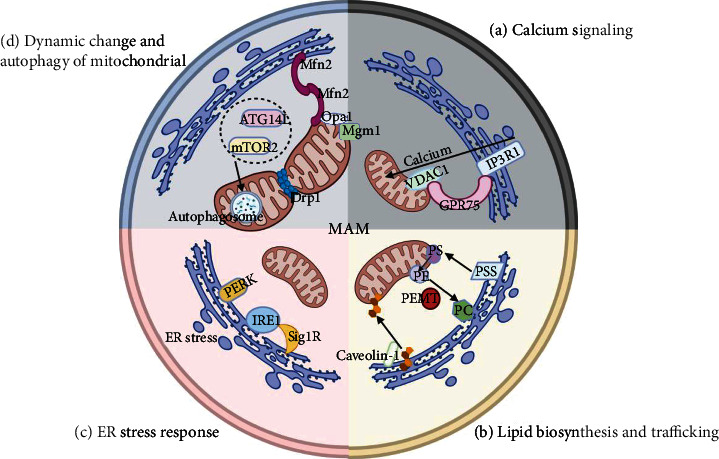
The functions of the mitochondrial-associated ER membrane. (a) Calcium signaling. Calcium can be transferred via the IP3R1 protein complex. VDAC1 serves as a calcium uptake channel. GRP75 can connect the two channel complexes mentioned above, which has also been designated the VDAC1/GRP75/IP3R1 axis. (b) Lipid biosynthesis and trafficking. PS is synthesized by PSS in the MAM and then transferred to mitochondria for further conversion to PE. PE can be delivered back to the MAM and converted into PC by PEMT. In addition, caveolin-1 participates in the transfer of cholesterol in the MAM. (c) ER stress response. The UPR is activated via IRE1 and PERK. Sig1R in the MAM can stabilize IRE1. (d) Mitochondrial dynamic changes and autophagy. The fusion and division of mitochondria are controlled by the dynamin-related GTPase Mgm1. The fusion of the MOM is mediated by Mfn1 and Mfn2. The fusion of the MIM is regulated by Opa1 and Mgm1. Mitochondrial division is controlled by Drp1. The recruitment of the preautophagosome marker ATG14L located in the MAM can trigger the formation of autophagosomes. Drp1 and ATG14L further enhance the enrichment of autophagy-related proteins in the MAM. As a main inducer of autophagy in the MAM, mTORC2 regulates the integrity of the MAM. Note: ATG14L, autophagy-related 14-like; FACL4, fatty acid CoA ligase 4; Drp1, dynamin-related protein 1; GRP75, chaperone 75 kDa glucose-regulated protein; ER, endoplasmic reticulum; IP3R, inositol-1,4,5-triphosphate receptor; MAM, mitochondria-associated ER membrane; IMM, inner mitochondrial membrane; Mfn, mitofusin; IRE1, inositol-requiring enzyme 1; mTORC2, mammalian target of rapamycin complex 2; Opa1, optic atrophy protein 1; PE, phosphatidylethanolamine; PEMT2, phosphatidyl ethanolamine methyltransferase 2; PS, phosphatidylserine; PSS, PS synthase; Sig 1R, sigma 1 receptor; VDAC1, voltage-dependent anion-selective channel protein 1; TRPV1, transient receptor potential cation channel subfamily V member 1; UPR, unfolded protein reaction; TRPM8, transient receptor potential melastatin 8.

MAM plays an important role in cellular activities. A growing number of findings support its participation in calcium signaling, lipid biosynthesis and trafficking, the ER stress response, dynamic changes and mitochondrial autophagy (Figure 3).

### 4.1. Calcium Signaling

Calcium homeostasis is of vital importance for cellular activities. Dysregulated calcium levels contribute to several physiological disorders and subsequently to cell death. Mitochondria and the ER play significant roles in calcium homeostasis. Ordinarily, calcium is stored in the ER. Upon cellular stimulation, calcium is released from the ER to the cytoplasm, where it is taken up by the mitochondria.

As the connecting structure, the MAM plays an important role in calcium signaling [47–49]. First, it was revealed that the structural integrity of the MAM can contribute to the maintenance of mitochondrial calcium homeostasis. Second, the microenvironment provided by the MAM is important for mitochondria to take up calcium. Third, some molecules that can regulate calcium signaling are located in or recruited to the MAM.

The transport of calcium from the ER to the mitochondria is controlled by several proteins located in the MAM. Inositol-1,4,5-triphosphate receptor type 1 (IP3R1) is the acknowledged channel for the release of calcium from the ER. In the outer mitochondrial membrane (OMM), voltage-dependent anion-selective channel protein 1 (VDAC1) serves as a channel for the uptake of calcium. Finally, the chaperone 75 kDa glucose-regulated protein (GRP75) can connect the two channel complexes mentioned above to form what is known as the VDAC1/GRP75/IP3R1 axis [50].

Recently, a new family of transient receptor potential melastatin 8 (TRPM8) channel isoforms located in the MAM was identified that acts as an ER calcium release channel [30]. Activation of TRPM8 appears useful for restricting cytosolic calcium signaling in the cardiovascular system [51]. Whether there are any other calcium channels in the MAM is unknown.

Overall, MAMs can affect calcium transfer from the ER, after which calcium migrates into the mitochondria via the mitochondrial calcium uniporter.

### 4.2. Lipid Biosynthesis and Trafficking

Lipids are important components of cell membranes, participating in energy storage, signal molecule transduction, and the synthesis of physiologically active substances. The synthesis of most lipids occurs in the ER, whereas several modifications of lipids occur in the mitochondria. The MAM participates in lipid transfer from the ER to the mitochondria.

Phosphatidylserine (PS) is synthesized by phosphatidyl serine synthase 1 (PSS1) and PSS2 in the MAM [52, 53]. Phosphatidylserine is then transferred to the mitochondria, decarboxylated and transformed into phosphatidylethanolamine (PE) [54]. In addition, PE can be delivered to MAMs and further transformed into phosphatidylcholine (PC) by PE-N-methyltransferase (PEMT) [55].

Additionally, MAM is involved in the metabolism of cholesterol. Cholesterol is composited in the ER and migrates into the mitochondria for conversion to pregnenolone. After the synthesis of cholesterol, caveolin-1 is inserted into the ER and participates in the delivery of cholesterol [36, 56]. The MAMs participate in the transport between the ER and the mitochondria by providing several enzymes involved in lipid biosynthesis and transport such as fatty acid CoA ligase 4 (FACL4), acetyl-CoA acetyltransferase 1 (ACAT1) and diacylglycerol-O-acyltransferase 2 (DGAT2). The enzyme FACL4 currently serves as a reliable MAM marker [22].

Overall, the MAM is involved in lipid metabolism by synthesizing and transporting lipids.

### 4.3. ER Stress Response

ER homeostasis is of vital importance for cellular activities. Unfolded protein reaction (UPR), an ER stress response, has been observed in DN. The activated UPR triggers three main components: inositol-requiring enzyme 1*α* (IRE1*α*), protein kinase RNA-like kinase (PERK) and activating transcription factor 6 (ATF6). A previous study reported that without PERK, ER stress-induced apoptosis is weakened due to a reduction in the MAM [57, 58]. In addition, IRE1 in the MAM can determine the effectiveness of IP3R, which contributes to the transfer of calcium from the ER to the mitochondria.

A variety of chaperone proteins related to protein folding are located in the MAM such as sigma 1 receptor (Sig1R) [38, 59], calnexin (CNX) [39], and calreticulin (CRT) [40]. The expression of Sig1R is increased when the PERK pathway is activated [60]. The protein Sig1R can inhibit caspase-4 activation and subsequently plays a protective role under conditions of ER stress. Calnexin and CRT are involved in calcium transport under conditions of ER stress.

Mild activation of ER stress might be beneficial, whereas excessive ER stress leads to cell death. Targeting maladaptive ER stress might help to rescue the development of DN [61]. In summary, some enzymes, molecules and chaperone proteins related to ER stress are located in the MAM. Thus, the MAM participates in the ER stress response.

### 4.4. Dynamic Change and Autophagy in Mitochondria

As dynamic organelles, mitochondria continuously undergo fission and fusion and move along the cytoskeleton. Mitochondrial damage leads to cardiac ischemia/reperfusion (I/R) injury and acute renal injury. Wang et al. demonstrated that Bax inhibitor-1 (BI1) could serve as a master regulator of renal tubular function by sustaining mitochondrial localization of prohibitin 2 (PHB2) [62]. In general, an excess nutrient supply leads to mitochondrial division, whereas starvation leads to mitochondrial fusion. Mitochondrial fusion and division are controlled by dynamin-related GTPases. Fusion of the OMM is mediated by Mfn1 and Mfn2 [63, 64]. Fusion of the mitochondrial inner membrane (MIM) is regulated by optic atrophy protein 1 (Opa1) and Mgm1 [65, 66]. In addition, mitochondrial division is controlled by dynamin-related protein 1 (Drp1) [67]. Thus, the dynamin-related GTPase located in the MAM can regulate dynamic changes in the mitochondria.

Autophagy is considered an intracellular degradation process. The participation of mitochondria in autophagy was first observed by the team of Hailey et al. in 2010, who showed that mitochondrial-localized cytochrome b5 is transferred from mitochondria to autophagosomes upon starvation [68]. Hamasaki et al. revealed that mitochondria are involved in the formation of autophagosomes, and isolation membranes are formed in the MAM [69]. The recruitment of the preautophagosome marker autophagy-related 14-like (ATG14L), which is located in the MAM, can trigger the formation of autophagosomes. Drp1 and ATG14L further enhance the enrichment of autophagy-related proteins in the MAM [70]. As a main inducer of autophagy in the MAM, mTORC2 regulates the integrity of the MAM [71].

The MAMs can affect calcium transfer from the ER to the mitochondria. Dysregulated calcium signaling leads to impaired mitochondrial integrity. In addition, MAMs control dynamic changes and autophagy in mitochondria via small molecules such as Mfn, Drp and Opa, which subsequently govern mitochondrial integrity.

The MAMs serve as crucial regulators to maintain the homeostasis of cellular activities. Altered MAM integrity contributes to insulin resistance via the disturbance of lipid transfer, mitochondrial dysfunction, and impaired mitochondrial dynamics and mitophagy. Their dysregulation leads to impaired secretory function and mass of *β* cells. Moreover, improved integrity of the MAM might be associated with enhanced insulin sensitivity [72].

## 5. The Role of the MAM in DN

**Figure 4:**
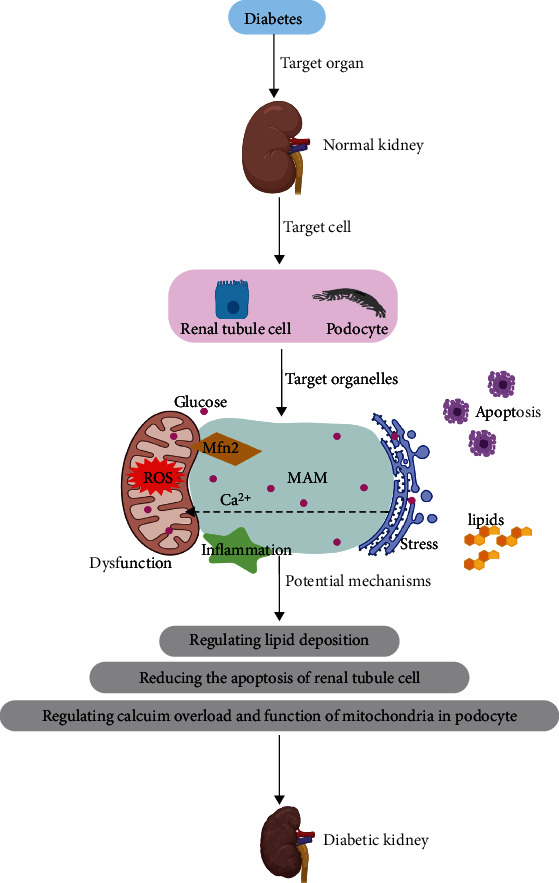
The role of the mitochondrial-associated ER membrane in diabetic nephropathy. The content and functions of MAM are changed under the conditions of DN. A dysfunctional MAM accounts for the development of DN. Note: ER, endoplasmic reticulum; DN, diabetic nephropathy; MAM, mitochondria-associated ER membrane.

As a microvascular complication, DN has become the leading cause of ESRD worldwide. Previously, Huang et al. summarized that phosphatase and tensin homolog (PTEN)-induced kinase 1 (PINK1) could regulate the function of mitochondria in DN, and targeting PINK1 might be a potential therapeutic strategy [73]. Currently, several studies have focused on MAM involvement in DN, which are summarized as follows (Figure 4):

### 5.1. Regulating Lipid Deposition

Previous studies have proven that high glucose triggers lipid disorders and deposition in the kidneys of animals [7, 74]. An increased accumulation of lipids in renal tissue accelerates the pathological changes in DN. In addition, lipid deposition in the kidney contributes to insulin resistance and enhances reactive oxygen species (ROS) production, the inflammatory response, and ER stress, which further accelerate the progression of renal damage in DN [75]. Yang et al. observed increased lipid deposition and damaged integrity of the MAM in kidneys of patients with DN [11]. They found a significantly negative association between the MAM and the serum levels of lipids, the renal accumulation of lipids, and decreased renal function. Interestingly, they observed downregulated expression of MAM-control proteins in different stages of DN. HK-2 cells incubated with high glucose exhibit impaired integrity of MAMs and enhanced lipid accumulation and apoptosis, which are alleviated by recovery of MAM integrity.

In addition, the crucial role of MAMs in ROS production should be mentioned. Reactive oxygen species could be produced in the mitochondria and ER. Excessive amounts of ROS are harmful. The MAMs serve as regulators of ROS synthesis and targets of oxidative damage. The MAM facilitates mitochondrial calcium uptake from the ER. The influx of calcium to the mitochondrial matrix affects the function of the mitochondria and ultimately ROS production [76].

Taken together, the destruction of the integrity of the renal MAM leads to renal lipid deposition and renal damage. However, the special mechanism by which the impaired integrity of MAMs contributes to renal lipid accumulation remains unclear. In vivo and in vitro experiments are encouraged to clarify this mechanism.

### 5.2. Reducing Apoptosis of Renal Tubule Cells

Apoptosis is programmed cell death, which is characterized by cell surface blebbing, volume reduction, internucleosomal cleavage of DNA and the formation of apoptotic bodies. Previous studies have suggested that the activation of cellular signal transduction contributes to apoptosis and that apoptosis plays a role in the development of DN [77–80]. Through detailed study, scholars have found that the MAM is involved in the induction of apoptosis in renal tubular epithelial cells in DN.

The biopsy of kidneys from patients with DN has revealed that enhanced apoptosis occurs in renal tubular epithelial cells [78, 81]. A detailed study by Yang et al. found increased renal apoptosis and tubulointerstitial fibrosis in patients with DN and in STZ-induced diabetic mice that was positively correlated with renal damage [82]. In addition, DsbA-L can inhibit apoptosis while maintaining MAM integrity and Mfn2 expression, which subsequently ameliorates renal damage in animals with DN and in high glucose-incubated HK-2 cells. Thus, these results provide momentum for investigating agonists of the MAM and DsbA-L as potential therapeutic agents to help regulate apoptosis, leading to the inhibition of DN progression.

### 5.3. Regulating Calcium Overload and the Function of Mitochondria in Podocytes

Appropriate mitochondrial calcium levels maintain normal oxidative respiration and ATP production by mediating the activity of TCA cycle rate-limiting enzymes and the ATP synthase [83–85]. The disturbance of mitochondrial calcium homeostasis, such as calcium overload, contributes to enhanced oxidative stress, apoptosis and inflammation [86–90].

Emerging evidence has proven that the MAM is involved in insulin and glucose signaling and plays a vital role in controlling glucose metabolism [72, 91, 92]. As a consequence, enhanced formation of the MAM serves as a major target promoting mitochondrial dysfunction in DN. Wei et al. reported that the hyperglycemic status-induced augmented formation of the MAM in podocytes is the indispensable step leading to calcium overload and renal injury in db/db- or STZ-induced diabetic mice [93]. The activation of calcium channel transient receptor potential cation channel subfamily V member 1 (TRPV1) by dietary capsaicin can promote AMPK activation, thus decreasing MAM-regulated mitochondrial calcium overload and dysfunction in podocytes [93].

In addition, the functional role of the MAM in inflammation has raised great concern. Systemic and local low-grade inflammation and the release of proinflammatory cytokines are common features in the development and progression of DN [94]. A recent study proved the relationship between NLRP3 inflammasome formation and the MAM. Resting NLPR3 is located in the ER, whereas the activated NLRP3 inflammasome is located in the MAM. Mitochondrial ROS induce the activation of NLRP3 via VDAC. VDAC facilitates the uptake of calcium into the mitochondria from the MAM to enhance mitochondrial activity [95, 96]. Excessive mitochondrial calcium influx leads to calcium overload. Calcium communication links MAM and NLRP3 inflammasome activation in DN [93]. Calcium overload is associated with mitochondrial destabilization, thus triggering NLRP3 inflammasome activation [97]. Further studies are encouraged to identify the underlying mechanisms by which the MAM regulates the inflammatory response.

In summary, the importance of the MAM in the development of DN has been gradually recognized. Renal tubular epithelial cells and podocytes are the main target cells for dysfunction of the MAM. In patients with DN and in diabetic animals, the integrity of the MAM is impaired. Damaged MAMs result in lipid deposition and increasing apoptosis of renal tubular cells, promoting calcium overload and disturbing podocytes. Gene disruption of DsbA-L and activation of the TRPV1 channel alleviate renal damage in diabetic nephrology in vivo and in vitro by regulating the MAM. More studies are encouraged to explore the role and mechanism of the MAM in DN.

## 6. Conclusions

The MAM is a highly plastic structure with structural parameters. Several enzymes, molecules, and chaperones are located in the MAM. Thus, under pathological conditions, such as hyperglycemia, ER stress, and inflammation, enhanced formation of the MAM is involved in cellular activities.

The function of the MAM was summarized in this article. However, additional details need further study. First, the role of the ER and mitochondria in the pathophysiology of DN has aroused great interest, but the role of the MAM in DN has not received sufficient attention. Second, the MAM not only initiates cellular activities associated with mitochondria and the ER but also provides a place where inflammasome and mitochondrial fission occurs by recruiting master effector proteins and signaling molecules. Third, emerging proteins located at the MAM are involved in controlling the function of the MAM. However, their functions in DN are not fully understood and require further research.

## 7. Future Perspectives

At present, there remain three obstacles to determining the role of the MAM in DN. First, although the isolation of purified MAM has been studied and continuously improved, it is nevertheless important to bear in mind that the MAM is not an isolated compartment but a dynamic and transient lipid raft domain in the ER, defined by the activities located within it. Our existing protocols for isolating the MAM require a large number of materials and cannot capture its dynamic nature. In addition, some protein modifications or interactions might be affected due to the lengthy procedure.

Second, the effects of MAM formation in different cell types have not been identified and may show different results. For the kidney, the nephron includes several cell types such as tubular epithelial cells, podocytes, glomerular endothelial cells and mesangial cells. It is important to explore the roles of MAMs in a cell-specific manner. An improved understanding will help to develop new therapeutic targets for MAM-related diseases.

Third, regulating the communication of the MAM is a double-edged sword. For example, enhanced formation of the MAM increases mitochondrial calcium uptake and calcium overload, leading to mitochondrial swelling and cell apoptosis. Mitochondrial calcium levels need to be controlled within a certain range. Therefore, precisely regulating the formation and function of the MAM might be a promising target for future studies.

We believe that ongoing studies will shed light on the role and specific mechanism of the MAM in DN. A better understanding of this axis will provide clues for more advanced therapeutic targets in DN.

## Figures and Tables

**Table 1:** The main components of the MAM.

Number	Classification	Components	References
1	Calcium channels in the outer membrane of the ER and mitochondria, voltage-dependent anion channels	TRPM8, VDAC, IP3R1, GRP75	[30, 31]
2	Enzymes for the synthesis and transfer of lipids	PSS1, PSS2, PEMT, caveolin-1, FACL4, ACAT1, DGAT2	[22, 32–37]
3	Chaperones	Sig1R, CNX, CRT	[38–40]
4	Redox reactive enzymes involved in the ER	ERO 1*α*	[41]
5	Mitochondrial rho GTPases 1 and mitofusin 2	MiRo 1, Mfn, FUNDC1, Drp	[42–46]

Note: ACAT, acetyl-CoA acetyltransferase 1; ATG14L, autophagy-related 14-like; CNX, Calnexin; CRT, calreticulin; DGAT2, diacylglycerol-O-acyltransferase 2; FACL4, fatty acid CoA ligase 4; Drp1, dynamin-related protein 1; ER, endoplasmic reticulum; ERO 1*α*, ER oxidoreductin-1*α*; PEMT, phosphatidyl ethanolamine methyltransferase; GRP75, chaperone 75 kDa glucose- regulated protein; IP3R, inositol-1,4,5-triphosphate receptor; IRE1, inositol-requiring enzyme 1; Mfn, mitofusin 2; TRPM8, transient receptor potential melastatine 8; PERK, protein kinase-like ER kinase; PS, phosphatidylserine; PSS, PS synthase; Sig1R, Sigma 1 receptor; VDAC1, voltage-dependent anion-selective channel protein 1.

## Data Availability

The data used to support the findings of this study are available from the corresponding author upon request.

## Abbreviations

ACAT: Acetyl-CoA acetyltransferase 1
AMPK: Adenosine 5'-monophosphate (AMP) activated protein kinase
ATG14L: Autophagy-related 14-like
ATP: Adenosine triphosphate
ATF6: Activating transcription factor 6
CNX: Calnexin
CRT: Calreticulin
Drp1: Dynamin-related protein 1
DGAT2: Diacylglycerol-O-acyltransferase 2
DM: Diabetes mellitus
DN: Diabetic nephropathy
ESRD: End stage renal disease
ER: Endoplasmic reticulum
FACL4: Fatty acid CoA ligase 4
GRP75: Chaperone 75 kDa glucose- regulated protein
IRE1: Inositol-requiring enzyme 1
IMM: Inner mitochondrial membrane
IP3R: Inositol-1,4,5-triphosphate receptor
MAM: Mitochondria-associated ER membrane
MFN2: Mitofusin 2
OMM: Outer mitochondrial membrane
mTORC2: Mammalian target of rapamycin complex 2
NLRP3: Pyrin domain-containing 3 protein
PEMT2: Phosphatidyl ethanolamine methyltransferase 2
Opa1: Optic atrophy protein 1
PC: Phosphatidycholine
PS: Phosphatidylserine
PSS: PS synthase
PE: Phosphatidylethanolamine
PERK: Protein kinase-like ER kinase
ROS: Reactive oxygen species
Sig1R: Sigma 1 receptor
STZ: Streptozocin
T1DM: Type 1 DM
T2DM: Type 2 DM
TRPM8: Transient receptor potential melastatin 8
TRPV1: Transient receptor potential cation channel subfamily V member 1
TCA: Tricarboxylic acid
UPR: Unfolded protein reaction
VDAC1: Voltage-dependent anion-selective channel protein 1.
